# SARS-CoV-2 Infection and Mitigation Efforts among Office Workers, Washington, DC, USA

**DOI:** 10.3201/eid2702.204529

**Published:** 2021-02

**Authors:** Samira Sami, Nga Vuong, Halie Miller, Rachael Priestley, Matthew Payne, Garrett Licata-Portentoso, Jan Drobeniuc, Lyle R. Petersen

**Affiliations:** Centers for Disease Control and Prevention, Atlanta, Georgia, USA (S. Sami, H. Miller, R. Priestley, J. Drobeniuc);; Centers for Disease Control and Prevention, Fort Collins, Colorado, USA (N. Vuong, L.R. Petersen);; Federal Emergency Management Agency, Washington, DC, USA (M. Payne, G. Licata-Portentoso)

**Keywords:** SARS-CoV-2, seroepidemiologic studies, emergency responders, Washington, DC, United States, 2019 novel coronavirus disease, severe acute respiratory syndrome coronavirus 2, coronavirus disease, COVID-19, worker safety, viruses, respiratory infections, zoonoses

## Abstract

Despite mitigation efforts, 2 coronavirus disease outbreaks were identified among office workers in Washington, DC. Moderate adherence to workplace mitigation efforts was reported in a serologic survey; activities outside of the workplace were associated with infection. Adherence to safety measures are critical for returning to work during the pandemic.

On March 19, 2020, the Federal Emergency Management Agency (FEMA) activated the National Response Coordination Center in Washington, DC, USA, in response to the coronavirus disease (COVID-19) pandemic. At that time, cases were rapidly increasing in Washington, DC; ≈200 cases had been reported since March 7. Although city officials ordered closure of nonessential businesses on March 24, FEMA remained open. To protect staff from severe acute respiratory syndrome coronavirus 2 (SARS-CoV-2) infection, all persons entering FEMA headquarters underwent symptom and temperature screening. On April 5, after a cluster of 6 epidemiologically linked cases was identified, additional mitigation efforts were implemented, including requiring face masks at all times, requiring that a distance of 6 feet be maintained between employees, and reducing occupancy in the open office space building from a daily average of 1,300 to 400 persons. 

To examine workplace and community factors associated with infection, we conducted a serologic survey of SARS-CoV-2 antibodies among staff who worked on site after the mitigation efforts had been implemented. To assess the effect of mitigation efforts in the workplace, we examined occupational case surveillance data.

Staff who worked in the FEMA building during April 1–22 were identified by using turnstile records and were invited by email to participate in a survey. Persons who had had symptoms of COVID-19 within 2 weeks of the survey were ineligible to participate. During April 23–29, consenting participants completed a self-administered, online questionnaire assessing demographics and potential community and workplace exposure to SARS-CoV-2, and blood samples were collected.

Blood samples were tested for SARS-CoV-2 IgG by using ELISA targeting the SARS-CoV-2 receptor-binding domain protein (*1*). Indeterminate test results or incomplete questionnaires resulted in the exclusion of 10 participants. Characteristics of seropositive and seronegative groups were compared by using the Fisher exact test, and 2-sided p values <0.05 were considered statistically significant. Reports of confirmed COVID-19 cases among staff who worked at FEMA headquarters during March–October 2020 were obtained from occupational health records. This activity was reviewed by the Centers for Disease Control and Prevention and deemed public health surveillance.

Of the 466 survey participants, 15 (3.2%) tested positive for SARS-CoV-2 antibodies. Seroprevalence did not vary by sex or age (Table). Of those who tested positive, 11 (73%) reported never having been tested for SARS-CoV-2 by nasal or throat swab, and 8 (53%) reported no symptoms suggestive of SARS-CoV-2 infection since January 15, 2020 (*2*). On average, participants had spent 20.5 (± 12.0 SD) days in the FEMA building since March 2020. We found no significant difference in workplace mitigation activities between seropositive and seronegative participants: 60.0% seropositive versus 60.5% seronegative participants used a face covering most of the time or always, 80.0% versus 76.3% maintained a distance of >6 feet from others most of the time or always, and 86.7% versus 91.1% washed their hands or used hand sanitizer >5 times per day. However, a higher, although not statistically significant, percentage of participants who shared a workspace were seropositive (13.3%) than seronegative (9.8%). The same was true for persons who spent >10 minutes <6 feet from someone who tested positive for SARS-CoV-2 in the FEMA building; 13.3% were seropositive and 10.2% were seronegative. A significantly higher percentage of seropositive participants lived with someone who had a confirmed positive test result for SARS-CoV-2 (13.3%) than those who were seronegative (0.7%). After the cancellation of nonessential gatherings on March 11, 60.0% of seropositive participants traveled by taxi or rideshare compared with 32.3% of seronegative participants who did not (p = 0.047).

**Table:** Characteristics and workplace and community exposure for SARS-CoV-2 infection among workers in the FEMA headquarters, by serologic testing results, Washington, DC, USA, April 2020*

Characteristic	SARS-CoV-2 result, no. (%)		p value†
	Positive (n = 15)	Negative (n = 451)	
Sex			
F	4 (26.7)	167 (37.0)	0.588
M	11 (73.3)	284 (63.0)	
Age group, y (n = 464)			
18–34	5 (33.3)	112 (24.9)	0.503
35–49	3 (20.0)	187 (41.5)	
50–64	7 (46.7)	139 (31.0)	
>65	0 (0.0)	11 (2.4)	
Mitigation activities in the workplace			
Wear a face cover (most or all the time)	9 (60.0)	273 (60.5)	0.298
Maintain a distance >6 feet from others (most or all the time)	12 (80.0)	344 (76.3)	1.000
Wash your hands or use hand sanitizer (>5 times daily)	13 (86.7)	411 (91.1)	0.147
Exposure to someone who tested positive for SARS-CoV-2 in the FEMA building			
Any face-to-face contact	2 (13.3)	51 (11.4)	0.224
>10 min within 6 feet	2 (13.3)	46 (10.2)	0.061
Shared workspace	2 (13.3)	44 (9.8)	0.062
Shared breakroom	1 (6.7)	30 (6.7)	0.286
Within 6 feet while coughing or sneezing	1 (6.7)	10 (2.2)	0.325
Exposure to household member with confirmed COVID-19	2 (13.3)	3 (0.7)	0.001
Community exposure during January 15–March 11			
Traveled by bus, train, or subway	8 (53.3)	318 (70.5)	0.161
Traveled by taxi or rideshare	9 (60.0)	290 (64.3)	0.787
Attended social gatherings of >50 persons	12 (80.0)	254 (56.3)	0.109
Visited a healthcare facility	8 (53.3)	150 (33.3)	0.162
Community exposure during March 12 through date of blood draw			
Traveled by bus, train, or subway	5 (33.3)	204 (45.2)	0.436
Traveled by taxi or rideshare	9 (60.0)	147 (32.6)	0.047
Attended social gatherings of >50 persons	2 (13.3)	55 (12.2)	0.704
Visited a healthcare facility	2 (13.3)	64 (14.2)	1.000

*COVID-19, coronavirus disease; FEMA, Federal Emergency Management Agency; SARS-CoV-2, severe acute respiratory syndrome coronavirus 2. †Fisher exact test for categorical variables.

By October 30, after mitigation efforts were implemented, 2 clusters of epidemiologically linked COVID-19 cases were identified: 4 cases among staff in cluster B and 5 cases in cluster D (Figure). We identified an additional 6 nonlinked cases among staff who worked in the FEMA building. Overall, 15 (71%) cases were linked to a cluster.

**Figure:**
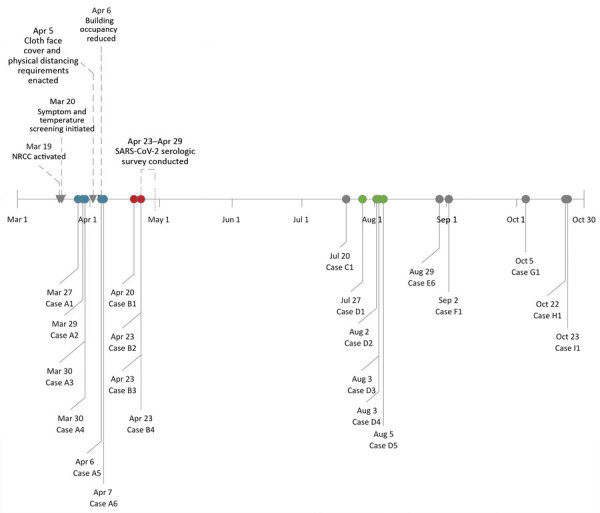
Coronavirus disease cases among workers in the Federal Emergency Management Agency, by case reporting date, and critical events, Washington, DC, USA, March–October 2020. Associated colors and A, B, and D indicate infection clusters. NRCC, National Response Coordination Center; SARS-CoV-2, severe acute respiratory syndrome coronavirus 2.

To our knowledge, evaluations of workplace SARS-CoV-2 mitigation strategies in office buildings have not been published. This study identified 2 factors outside of the workplace that are potentially associated with SARS-CoV-2 infection and transmission in the workplace (despite limited knowledge of whether infection occurred before or after potential exposure): residing with a household member with COVID-19 and using shared transportation. Although seroprevalence for SARS-CoV-2 antibodies was low among office workers, preventing workplace exposures to COVID-19 during March–April 2020 remained challenging. More than half of seropositive participants remained asymptomatic or were never tested for SARS-CoV-2, and 20%–40% of participants did not adhere to masking or physical distancing guidelines. This finding highlights the difficulties of adhering to mitigation efforts in the workplace and the importance of ensuring prevention efforts as persons return to work, such as engineering controls to reduce occupancy levels and modifying areas to maintain a distance of 6 feet between employees (*3*). Despite hazard controls implemented in the workplace, activities outside of work and noncompliance with mitigation efforts probably contributed to cases and small clusters of COVID-19 among office workers. However, seroprevalence remained at the same level as the overall 3.2% seroprevalence estimate for Washington, DC residents (*4*).
